# Classification of IHC Images of NATs With ResNet-FRP-LSTM for Predicting Survival Rates of Rectal Cancer Patients

**DOI:** 10.1109/JTEHM.2022.3229561

**Published:** 2022-12-15

**Authors:** Tuan D. Pham, Vinayakumar Ravi, Chuanwen Fan, Bin Luo, Xiao-Feng Sun

**Affiliations:** Center for Artificial IntelligencePrince Mohammad Bin Fahd University 125898 Khobar 31952 Saudi Arabia; Department of OncologyLinkoping University 58185 Linkoping Sweden; Department of Biomedical and Clinical SciencesLinkoping University 58185 Linkoping Sweden; Department of Gastrointestinal SurgerySichuan Provincial People’s Hospital Chengdu 610032 China

**Keywords:** Rectal cancer, 5-year survival prediction, artificial intelligence, deep learning, fuzzy recurrence plots

## Abstract

Background: Over a decade, tissues dissected adjacent to primary tumors have been considered “normal” or healthy samples (NATs). However, NATs have recently been discovered to be distinct from both tumorous and normal tissues. The ability to predict the survival rate of cancer patients using NATs can open a new door to selecting optimal treatments for cancer and discovering biomarkers. Methods: This paper introduces an artificial intelligence (AI) approach that uses NATs for predicting the 5-year survival of pre-operative radiotherapy patients with rectal cancer. The new approach combines pre-trained deep learning, nonlinear dynamics, and long short-term memory to classify immunohistochemical images of RhoB protein expression on NATs. Results: Ten-fold cross-validation results show 88% accuracy of prediction obtained from the new approach, which is also higher than those provided from baseline methods. Conclusion: Preliminary results not only add objective evidence to recent findings of NATs’ molecular characteristics using state-of-the-art AI methods, but also contribute to the discovery of RhoB expression on NATs in rectal-cancer patients. Clinical impact: The ability to predict the survival rate of cancer patients is extremely important for clinical decision-making. The proposed AI tool is promising for assisting oncologists in their treatments of rectal cancer patients.

## I. Introduction

Immunohistochemistry (IHC) is an image-staining technique for visualizing antigens (proteins) in cells of tissue by employing fluorescence microscopy. IHC images are used for detecting abnormal cells such as those found in malignant tumors [1], discovering biomarkers of diseases, and validating candidate drug efficacy [2]. Techniques for image analysis of IHC staining have been well recognized as an important role in digital pathology [3], [4]. Furthermore, the combination of artificial intelligence and image analysis techniques have recently been reported to significantly enable new discoveries in oncology [5], including colorectal cancer [6], [7], [8], and advance research in pathology and medicine [9], [10].

Rectal cancer is a disease in which cancerous cells uncontrollably develop in the tissues of the rectum. The overall 5-year survival rate for patients with rectal cancer is about 63% [11]. The American Cancer Society reported that 5-year (relative) survival rates for rectal cancer are 90%, 73%, and 17% for localized, regional, and distant SEER (Surveillance, Epidemiology, and End Results) staging, respectively; and 67% for the combination of all SEER stages [12].

Over the past decade, tissues dissected adjacent to tumors are considered as normal, and so-called normal tissues adjacent to tumors (NATs) [13], [14]. Recent findings have shown that NATs’ molecular characteristics are quite different from both healthy tissues and tumors [15]. NATs are essential for understanding recurrent tumors and selecting optimal surgical strategies [16]. However, it is suggested to use computational methods to independently confirm the distinct molecular patterns of NATs [15].

In an attempt to discover the prognosis of breast cancer, a study investigated the use of gene expression profiles in NATs for survival prediction of breast cancer patients [17]. The study was carried out by analyzing the genetic characteristics of NATs obtained from The Cancer Genome Atlas, and a particular gene expression profile in normal tissues around breast tumors was found to be associated with the 10-year survival rate for breast cancer women with estrogen receptor-positive cases, which accounts for about 70% among patients with breast cancer. It was reported in a recent study that NATs provide important molecular information, which can be used to differentiate tumors of being more and less aggressive [18]. The study investigated 52 NAT samples of prostate cancer patients obtained from the Cancer Genome Atlas to develop a genetic model for the prediction of recurrence-free survival. The survival-prediction power of NATs was confirmed with an independent cohort, suggesting distinct prognostic mechanisms between NATs and tumor tissues. Similarly, another study of NATs for survival prediction in hepatocellular carcinoma (HCC), which is the most frequent primary liver cancer, reported the critical function of Hippo signaling in normal tissues adjacent to HCC with respect to the cancer development [14]. The study constructed a model of Hippo-related gene expression profiles in NATs for predicting the prognosis of patients with HCC. The univariate Cox regression analysis identified 14 genes that can be used for the survival prediction in HCC with an area under the receiver operating characteristic curve of 0.75.

This paper reports the first attempt to investigate the power of RhoB expression in images of rectal-cancer NATs with deep learning and nonlinear time-series analysis. Using IHC images of RhoB protein expression on rectal-cancer NATs, this study combines the methods of pre-trained convolutional neural networks (CNNs), nonlinear dynamics, and long short-term memory (LSTM) for classifying IHC images of the NATs. Pretrained CNNs are utilized for extracting deep features of IHC images. The method of fuzzy recurrence plots (FRPs) [19] developed for studying nonlinear dynamics is then applied to transform long CNN-based feature vectors into FRPs, which are short multidimensional time series. Finally, an LSTM network is used to learn these FRPs for time-series classification to predict the life expectancy of two cohorts of rectal-cancer patients who took pre-operative radiotherapy and lived more or less than 5 years after surgery.

The motivation for extracting features of the IHC images with pretrained CNNs and constructing FRPs of these extracted features is based on the following aspects of data analysis for machine learning. Firstly, extracting deep features of new data from pre-trained CNNs has been reported very useful in terms of robustness and computational advantage for the classification of complex biomedical images [20], [21], [22], [23]. Secondly, the transformation of flattened deep image features into FRPs is expected to enhance the power of machine learning as the spatial-temporal content of the original IHC data can be captured by this method of nonlinear dynamics. Finally, the transformed spatial-temporal features represented by the FRPs can be used to provide multiple feature dimensions in a much shorter time series for increasing the sequential learning power of the LSTM. Thus, the combination of two state-of-the-art AI approaches (CNN and LSTM models) by means of the FRP construction is unique for predicting survival rates in patients with rectal cancer, which is the major contribution of this study.

The rest of this paper is organized as follows. Section II describes methods employed for deep learning and classification of IHC-images to predict the 5-year survival of rectal cancer patients. Section III presents and discusses results. Finally, Section IV provides concluding remarks on the finding.

## II. Methods

### A. Feature Extraction From Pre-Trained CNNs

CNNs are deep neural networks, which consist of a series of three main types of layers known as convolutional, pooling, and fully-connected layers. A feature map $G$ of an input image $I$ can be produced using the convolutional process as follows.

\begin{align*} G(x,y)=&W \circledast I(x,y) \\=&\sum _{dx=-\alpha }^{\alpha } \sum _{dy=-\beta }^{\beta } W(dx,dy) I(x+dx,y+dy), \\{}\tag{1}\end{align*}

where $W$ is a filter kernel.

To avoid negative values of the feature map, the rectified linear unit (ReLU) [24], denoted as $L$, is then applied to $G$. This nonlinear function is defined as

\begin{align*} L(\omega) = \begin{cases} 0, & \text {for} \: \: \omega < 0 \\ \omega, & \text {for} \: \: \omega \geq 0 \end{cases}\tag{2}\end{align*}

There is no single theoretical method for selecting an optimal activation function for hidden layers of a deep net to learn on a particular dataset. The ReLU is the most widely used activation function because it can effectively address the vanishing gradient problem when training a deep neural network [25]. However, the disadvantage of the ReLU is that, in some given datasets, it transforms many neurons with negative values into zeros, resulting in many dead neurons that will never be updated. To resolve the dead-neuron problem, several extended versions of ReLU were developed to avoid the nullification of negative-value neurons, including leaky ReLU (LReLU) [26], parametric ReLU (PReLU) [27], randomized leaky ReLU (RLReLU) [28], and S-shaped ReLU (SReLU) [29]. Mathematical descriptions and applications of these ReLU variants were reviewed in [25].

Next the pooling operator is applied to the rectified feature map in an attempt to produce down-sampling. The most widely used pooling for CNNs is the maximum operator, denoted as $D_{\max }$, which operates on a collection of $M$ pooling regions $O^{p}$, $p = 1, {\dots }, M$, as

\begin{equation*} D_{\max } (O^{p}) = \max _{1 \leq \kappa \leq m \times m} (o_{\kappa }^{p}).\tag{3}\end{equation*}

where $o_{\kappa }^{p} \in O^{p} = (o_{1}^{p}, {\dots }, o_{m \times m}^{p})$, and $m \times m$ is the number of pixels in a pooling region.

After a series of operations on convolution and pooling, the final feature map is flattened and fed into the fully-connected (hidden) layer followed by the softmax layer for computing the classification probability distribution over a set of objects.

In this study, the IHC images were input into a pre-trained CNN, which went through the network learning. Finally, the flattened final feature map was then extracted for classification by other machine-learning models. In fact, pretrained CNNs that were trained on the ImageNet database [30] for learning and extracting complex features from different types of images have been often used to classify new objects, where the sample size in the new task is small [6], [20]. Such utilization of a pretrained network is known as transfer learning, which offers certain advantages for feature extraction of complex medical images for pattern classification having mentioned earlier. Furthermore, the extraction of IHC image features from pre-trained CNNs has been utilized for pattern classification, because it offers the fastest procedure to take advantage of the power of deep learning [31]. This type of feature extraction is time-saving, because it requires only a single pass through the data and avoids the need for network training.

Three pre-trained CNNs that were used in this study for extracting features of the IHC images are briefly described as follows.

• NASNet-Large is a variant of the Neural Architecture Search Network (NASNet) models [32]. This net was designed to consist of normal and reduction cells to carry out search space, search strategy, and performance estimation to identify the best algorithm in order to achieve the best optimal performance over a certain piece of work. NASNet-Large has the image input size of $331 \times 331 \times 3$ pixels.
• DenseNet-201 is a pretrained CNN, which is a variant of the DenseNet [33]. DenseNet-201 has 201 layers. This network has an architecture that allows collective information received from the prior layers can reduce the number of channels, making the network become dense. The size of an input image to DenseNet-201 is of $224 \times 224 \times 3$ pixels.
• ResNet-101 is one of the ResNet (Residual Networks) family [34]. It is a pretrained CNN. This network requires the input image size of $224 \times 224 \times 3$ pixels. This network learns the residual functions with reference to the layer inputs instead of the signals and stacks residual blocks on top of each other to form a network of 101 layers deep. Because of such architecture, it is relatively easier to optimize these networks, which are expected to increase classification accuracy by increasing the network depth.

As pre-trained CNNs built hierarchical representations of input images, where deeper CNN layers generated higher-level features, which were constructed based on lower-level features obtained from earlier layers. Feature extraction of the IHC images from pre-trained CNNs was taken from the fully-connected layers that combined features of the input images over all spatial locations. This layer selection for extracting features from pre-trained CNNs was reported as being effective for classification tasks by several previous studies [35], [36], [37], [38],

Because the above three pre-trained networks require input images of different sizes ( $331 \times 331 \times 3$ for NASNet-Large and $224 \times 224 \times 3$ for DenseNet-201 and ResNet-101), all training and test IHC images were resized to the specified sizes before they were input to the pre-trained CNNs. NASNet-Large, DenseNet-201, and ResNet-101 are among popular pre-trained deep-learning models, and discussions on their computing designs and structures are widely available in AI literature reviews. Interested readers can refer to original developments reported in [32], [33], and [34] for detailed descriptions of the architectures of the pre-trained CNNs adopted in this study.

### B. FRPs of Pretrained CNN Features

Let ${\mathbf{g}} = (g_{1}, g_{2}, {\dots }, g_{N})$ be a vector of extracted image features flattened by a pretrained CNN. To significantly reduce the computational complexity of constructing an FRP of a long time series, ${\mathbf{g}}$ can be represented with a sequence of its cluster centers ${\mathbf x} = (x_{1}, x_{2}, {\dots }, x_{n})$, where $n \ll N$ by using the fuzzy $c$-means (FCM) algorithm [39]. To compute an FRP of ${\mathbf x}$, first a phase-space reconstruction from ${\mathbf x}$ is carried out using Takens’ time-delay embedding theorem [40], yielding

\begin{equation*} {\mathbf S} = (\mathbf {s}_{1}, \mathbf {s}_{2}, {\dots }, \mathbf {s}_{M}),\tag{4}\end{equation*}

where $M = n-(d-1)\tau $, $d$ is an embedding dimension, $\tau $ is a time delay, and

\begin{equation*} {\mathbf s}_{i} = (x_{i}, x_{i+\tau }, {\dots }, x_{i+(d-1)\tau }), \: i = 1, {\dots }, n-(d-1)\tau.\tag{5}\end{equation*}

Given a number of clusters $c$ and fuzzy exponent $m$, the FCM is then applied to divide ${\mathbf S}$ into $c$ groups, which are represented with a set of $c$ cluster centers $(\mathbf {v}_{1}, \mathbf {v}_{2}, {\dots }, \mathbf {v}_{c})$. The FCM also yields a matrix of fuzzy membership grades $\mu (\mathbf {s}_{i},\mathbf {v}_{k}) \in [{0, 1}]$, $i=1, {\dots }, M$, $k=1, {\dots }, c$, which express the similarity between $\mathbf {s}_{i}$ and $\mathbf {v}_{k}$. Finally, using the FCM results, an FRP, denoted as ${\mathbf F}$, is constructed as an $M \times M$ grayscale image that takes real values in [0, 1] as [19]

\begin{equation*} {\mathbf {F}}(i,j) = \mu (\mathbf {s}_{i},\mathbf {s}_{j}), \: i, j = 1, {\dots }, M, \tag{6}\end{equation*}

where $\mu (\mathbf {s}_{i},\mathbf {s}_{j})$, which is a fuzzy membership of similarity between $\mathbf {s}_{i}$ and $\mathbf {s}_{j}$, is determined using the following three properties.

1) Reflexivity:
\begin{equation*} \mu (\mathbf {s}_{i},\mathbf {s}_{i}) = 1, \: i=1, {\dots }, M.\tag{7}\end{equation*}
2) Symmetry:
\begin{equation*} \mu (\mathbf {s}_{i},\mathbf {v}_{k}) \!= \!\mu (\mathbf {v}_{k},\mathbf {s}_{i}), \: i \!= \!1, {\dots }, M, k \!= \!1, {\dots }, c.\tag{8}\end{equation*}
3) Transitivity:
\begin{align*} \mu (\mathbf {s}_{i},\mathbf {s}_{j}) = \max \left [{\min \{\mu (\mathbf {s}_{i},\mathbf {v}_{k}), \mu (\mathbf {v}_{k}, \mathbf {s}_{j})\} }\right], \\ i \neq j; k = 1, {\dots }, c.\tag{9}\end{align*}

The determination of $\mu (\mathbf {s}_{i},\mathbf {v}_{k})$, $i=1, {\dots }, M$, $k=1, {\dots }, c$, can be obtained by the FCM that tries to minimize the following objective function [39]:

\begin{equation*} F_{m} = \sum _{i=1}^{M}\sum _{k=1}^{c} [\mu (\mathbf {s}_{i},\mathbf {v}_{k})]^{m} \| {\mathbf s}_{i} - {\mathbf v}_{k} \|^{2},\tag{10}\end{equation*}

where $m \in [1, \infty$) is the weighting exponent, and $F_{m}$ is subject to

\begin{equation*} \sum _{k=1}^{c} \mu (\mathbf {s}_{i},\mathbf {v}_{k}) = 1;\: i=1, {\dots }, M.\tag{11}\end{equation*}

The objective function of the FCM is minimized by a numerical scheme, which iterates the update the fuzzy membership grades and cluster centers until the values converge. Given initialized fuzzy memberships, both ${\mathbf v}_{k}$ and $\mu (\mathbf {s}_{i},\mathbf {v}_{k})$ are iteratively updated as

\begin{align*} {\mathbf v}_{k}=&\frac {\sum _{i=1}^{N} (\mu (\mathbf {s}_{i},\mathbf {v}_{k}))^{m} \: {\mathbf s}_{i}}{\sum _{i=1}^{M} (\mu (\mathbf {s}_{i},\mathbf {v}_{k}))^{m}}. \tag{12}\\ \mu (\mathbf {s}_{i},\mathbf {v}_{k})=&\frac {1}{\sum _{j=1}^{c} \left ({\frac {\|{\mathbf s}_{i} - {\mathbf v}_{k}\|}{\|{\mathbf s}_{i} - {\mathbf v}_{j}\|} }\right)^{2/(m-1)} }; \tag{13}\end{align*}

The procedure for carrying out the FCM can be outlined as follows.

*FCM Algorithm*

1) Given data ${\mathbf S}$, number of clusters $c > 1$, weighting exponent $m$, threshold $\delta $, and maximum number of iterations $Q$.
2) Set index q = 1.
3) Initialize fuzzy-membership matrix ${\mathbf U}_{q}\,\,=$ [ $\mu _{q}(\mathbf {s}_{i},\mathbf {v}_{k})]$, $i = 1, {\dots }, M$; $k = 1, {\dots }, c$.
4) Compute $\mathbf {v}_{k}$, $k=1, {\dots }, c$ using Eq. (12).
5) Compute $\mu _{q+1}(\mathbf {s}_{i},\mathbf {v}_{k})$ using Eq. (13).
6) If $\lvert {\mathbf U}_{q} - {\mathbf U}_{q+1} \rvert \le \delta $ or $q = Q$, stop.
7) Otherwise, set $q = q+1$ and return to Step 4.

### C. Training LSTM Networks With FRPs

LSTM networks [41], [42] have the capability of handling the long-term dependency problem encountered by other recurrent neural networks. The long-term dependency induces a vanishing gradient, which becomes negligible to allow the updating of the network weights. Applications of LSTM networks have reportedly been useful for classifying physiological signals [43], [44], [45] and histopathological images [46].

Basically, the repeating block in an LSTM regulates how data at each time step are processed using four interacting components:

• Input gate, denoted as $u$, which controls the level of cell state update.
• Forget gate, denoted as $f$, which controls the level of cell state reset or forgetting.
• Cell candidate, denoted as $w$, which keeps and adds useful information to a cell state.
• Output gate, denoted as $e$, which controls the level of a cell state added to a hidden state.

LSTM learnable parameters of a layer consist of input weights ( $\mathbf I$), recurrent weights ( $\mathbf R$), and bias ( $\mathbf b$), which are mathematically expressed as

\begin{align*} {\mathbf I}=&[{\mathbf i}_{u}, {\mathbf i}_{f}, {\mathbf i}_{w}, {\mathbf i}_{e}]^{T}\tag{14}\\ {\mathbf R}=&[{\mathbf r}_{u}, {\mathbf r}_{f}, {\mathbf r}_{w}, {\mathbf r}_{e}]^{T}\tag{15}\\ {\mathbf b}=&[b_{u}, b_{f}, b_{w}, b_{e}]^{T}\tag{16}\end{align*}

The cell and hidden states at time step $t$ are denoted as ${\mathbf c}_{t}$ and ${\mathbf h}_{t}$, respectively, which are defined as

\begin{equation*} {\mathbf c}_{t} = f_{t} \circ {\mathbf c}_{t-1} + u_{t} \circ w_{t},\tag{17}\end{equation*}

where $\circ $ denotes the Hadamard product, and

\begin{equation*} {\mathbf h}_{t} = e_{t} \circ \sigma _{c}({\mathbf c}_{t}).\tag{18}\end{equation*}

At time $t$, input gate ( $u_{t}$), forget gate ( $f_{t}$), cell candidate ( $w_{t}$), and output gate ( $e_{t}$) are defined as

\begin{align*} u_{t}=&\sigma _{g} ({\mathbf i}_{u} {\mathbf z}_{t} + {\mathbf r}_{u} {\mathbf h}_{t-1} + {b}_{u}), \tag{19}\\ f_{t}=&\sigma _{g} ({\mathbf i}_{f} {\mathbf z}_{t} + {\mathbf r}_{f} {\mathbf h}_{t-1} + {b}_{f}), \tag{20}\\ w_{t}=&\sigma _{c} ({\mathbf i}_{w} {\mathbf z}_{t} + {\mathbf r}_{w} {\mathbf h}_{t-1} + {b}_{w}), \tag{21}\\ e_{t}=&\sigma _{g} ({\mathbf i}_{e} {\mathbf z}_{t} + {\mathbf r}_{e} {\mathbf h}_{t-1} + {b}_{e}),\tag{22}\end{align*}

in which ${\mathbf z}_{t}$ is the input feature vector at time $t$, $\sigma _{c}$ and $\sigma _{g}$ are state and gate activations usually expressed as the hyperbolic tangent and sigmoid functions, respectively.

In this study, an FRP, which is the final transformation of the associated IHC image described previously, is used as an input into an LSTM for the network training and classification. To be more explicit, $F(i,j)$, $i=1,2, {\dots }, M$, where $M$ is the number of features, is a sequence input into an LSTM, and $F(i,j)$, $j=1,2, {\dots }, M$, where $M$ is the number of time steps, is the input sequence of $M$ features at each time step $j$. More explicitly, $F(i,j)$, $i=1,2, {\dots }, M$, are the fuzzy membership grades of similarity computed to construct the FRP of an IHC image described in Section II-B, and used as a time series of multiple features for LSTM learning and prediction task.

**FIGURE 1.**
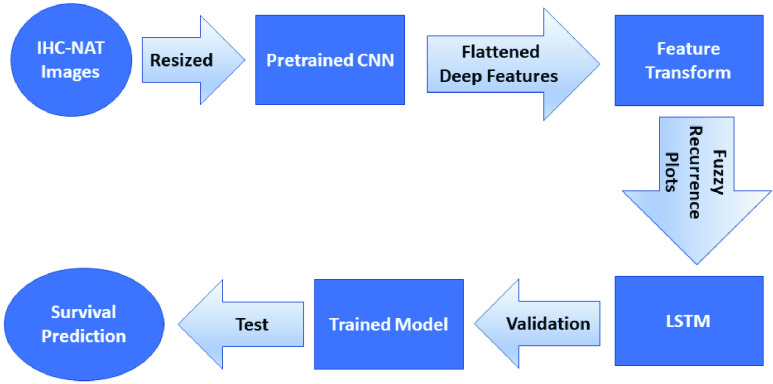
CNN-FRP-LSTM model for survival prediction in rectal cancer using IHC-NAT images.

Figure 1 shows the proposed procedure for extracting deep-learning features of IHC-NAT images from a pre-trained CNN, transforming the extracted deep-learning features into FRPs that are used for training and testing an LSTM network for the survival prediction.

**FIGURE 2.**
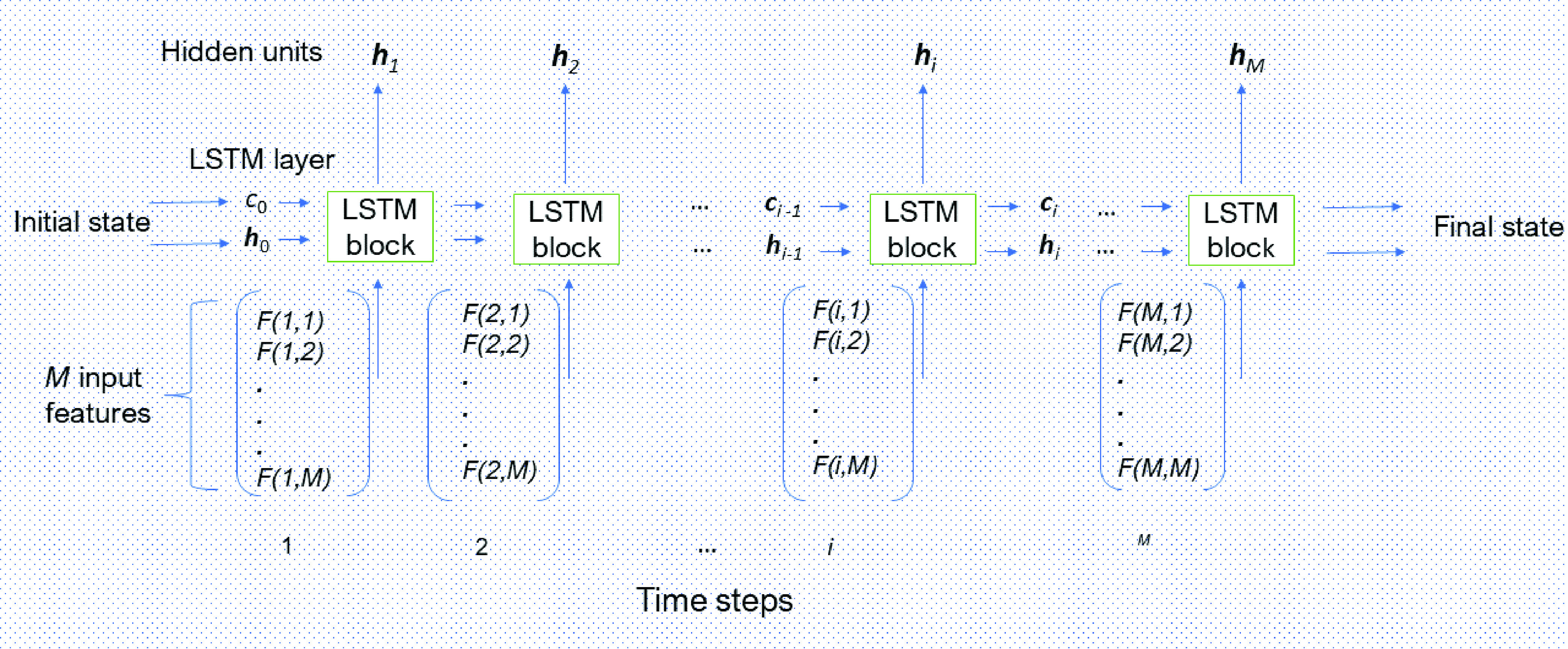
LSTM layer architecture with an FRP as input time series of multiple features.

Figure 2 shows how the flow of an FRP ${\mathbf F}$, which is presented as a time series of length $M$ with $M$ features, is processed by an LSTM layer. The first LSTM block takes information from the initial state of the network and the first time-step of the FRP to determine the first hidden and cell states. At time step $i$, the LSTM block uses the previous state pair of the network ( ${\mathbf h}_{i-1}$ and ${\mathbf c}_{i-1}$) and $M$ features of the time series (FRP) at time $i$ to compute ${\mathbf h}_{i}$ and ${\mathbf c}_{i}$. As a result, the current cell state contains information obtained from previous time steps, and at each time step, the LSTM layer adds important information to or removes redundant memory from the cell state during training. The network layer regulates these updates using the gate operators defined earlier.

### D. Performance Measures

To evaluate the performance of the proposed approach for survival prediction using IHC images of RhoB expression in rectal cancer biopsy, >5-year and < 5-year survival times are considered as positive and negative conditions, respectively. The numbers of samples of > and < 5 years of survival are denoted as $P$ and $N$, respectively. True positive ( $TP$) is the number of samples of > 5 years, which are correctly predicted as > 5 years; whereas true negative ( $TN$) is the number of samples of < 5 years being correctly predicted as < 5 years. False positive ( $FP$) is the number of samples of < 5 years, which are misclassified as > 5 years. False negative ( $FN$) is the number of samples of > 5 years, which are misclassified as < 5 years.

Equations used for calculating prediction/classification accuracy ( $ACC$), sensitivity or true positive rate ( $SEN$), specificity or true negative rate ( $SPE$), precision or positive predictive value ( $PRE$), and $F_{1}$ score are expressed in Table 1. For all measures, a higher value indicates better performance of the model in terms of the defined measure.

**TABLE 1:** Performance Measures of Prediction Models

$ACC$	$SEN$	$SPE$	$PRE$	$F_{1}$
$\dfrac {TP+TN}{P+N}$	$\dfrac {TP}{P}$	$\dfrac {TN}{N}$	$\dfrac {TP}{TP+FP}$	$\dfrac {2TP}{2TP+FP+FN}$

## III. Results

The proposed approach was tested using a subset of IHC images obtained from rectal-cancer patients. This subset consists of samples of RhoB-protein expression by IHC staining on tissues that were removed adjacent to primary tumors. The data were collected from two groups of rectal-cancer patients who had survival rates of less or more than 5 years. The study was conducted in accordance with the Declaration of Helsinki, and protocols approved by the Institutional Ethics Committee of Linkoping University (Dnr 2012-107-31 and Dnr 2014-79-31). These patients were participants included in the randomized Swedish Rectal Cancer Trial of preoperative radiotherapy between 1987 and 1990 [48]. There are 80 IHC samples of NATs, where 29 and 51 images are of less and more than 5-year disease-free survival, respectively. These samples were collected from rectal cancer patients who took pre-operative radiotherapy.

The layer named “global_average_pooling2d_2” (global average pooling features), which is before the fully connected layer, was used for feature extraction with NASNet-Large. Similarly, for DenseNet-201, the layer named “avg_pool” (global average pooling features), which is before the fully connected layer, was used for feature extraction. Likewise, for ResNet-101, the layer named “pool5” (global average pooling features), which is before the fully connected layer, was used for feature extraction. For classification using the three pre-trained CNNs, parameter specifications for the training were as follows. Minimum batch size = maximum number of epochs = 20, initial learning rate=0.0003, number of epochs for dropping the learning rate = 10, factor for dropping the learning rate =0.1, factor for $L_{2}$ regularization (weight decay) =0.0001, gradient threshold method $= L_{2}$ norm, gradient threshold $= \infty $, training and validation data were shuffled once before training, and networks were trained using stochastic gradient descent with momentum. Parameter fine-tuning was performed in this study for the transfer learning of the IHC data, because the three CNNs were pre-trained on different types of images [32], [33], [34]. Such parameter fine-tuning was necessary and found to be effective in several similar applications [6], [31], [49], [50].

Transfer learning of the resized IHC images resulted in flattened feature lengths of 4032, 1920, and 2048 obtained from NASNet-Large, DenseNet-201, and ResNet-101, respectively, at the selected deep layers described earlier. These flattened features were then used as the input for constructing the FRPs.

For model comparison, the same deep-learning parameters given above were used for the survival prediction performed directly by the three stand-alone pre trained CNNs (NASNet-Large, DenseNet-201, and ResNet-101). For another purpose of comparison, the linear support vector machine (SVM) model was adopted for the survival prediction using the same pretrained CNN-based features (NASNet-Large-SVM, DenseNet-201-SVM, and ResNet-101-SVM).

To further compare with other classifiers, 14 features from the gray-level co-occurrence matrices (GLCM) described in [51] were extracted from the grayscale IHC images and then used for the survival prediction by the linear SVM algorithm. In addition, the GooLeNet [52] and AlexNet [53], which are two widely used pre-trained CNNs, were also applied for classifying the IHC images for the survival prediction.

To illustrate the performance of the proposed approach, the same deep-learning features of the IHC images extracted from the pre-trained CNNs were adopted for constructing the FRPs that were modeled as time series of multiple features for the survival prediction by the LSTM-based classifier. For the use of LSTM, the bidirectional LSTM model was used for sequence classification, and its other parameter specifications are: maximum number of epochs = 300, minimum batch size=150, initial learning rate =0.01, and gradient threshold = 1.

For the classification by LSTM with the input as FRPs of features extracted from pre-trained CNN models, the number of clusters $c\,\,=13$ was used to compress the original CNN-based feature vectors into much shorter sequences of length $c$ by the FCM. To construct FRPs of the compressed sequences, $c\,\,=3$, fuzzy exponent $m\,\,=2$, embedding dimension $d\,\,=1$, and time delay $\tau \,\,=$ 1 were adopted in this study. In two cases for computing the FCM, threshold $\delta \,\,=0.00001$, and the maximum number of iterations $Q\,\,=100$. The construction of FRPs using the flattened features produced by each of the three pretrained CNNs and the specified FRP parameters resulted in output $13 \times 13$ matrices. These $13 \times 13$ FRPs were then used as the input into the LSTM to produce two outputs of scores for predicting the two survival times of < and > 5 years.

**FIGURE 3.**
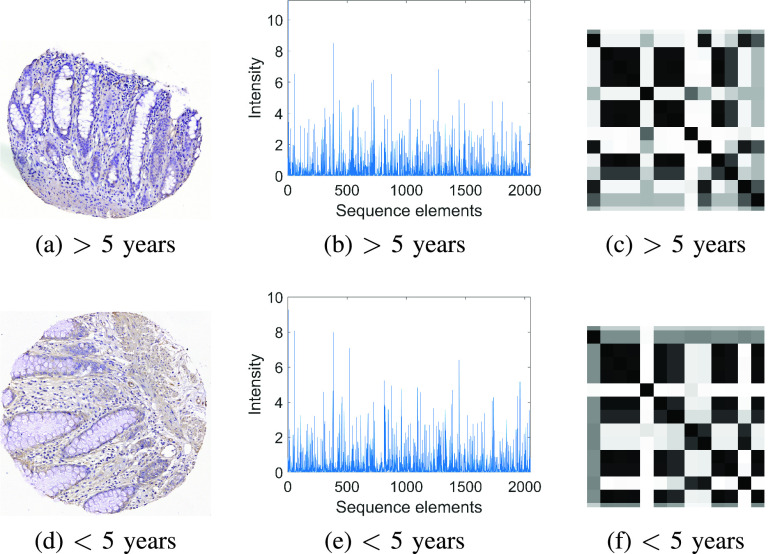
RhoB-expressed IHC images of tissues dissected adjacent to primary tumors of rectal cancer (a) & (d), associated ResNet-101 flattened features (b) & (e), and FRPs (c) & (f).

Figure 3 shows IHC images of RhoB expression on NATs of rectal-cancer patients who had < or > 5 years of survival after surgery, their feature vectors extracted from the ResNet101, and corresponding FRPs, respectively.

Table 2 shows the performance metrics based on 10-fold cross-validation obtained from different classification models, where NASNet-Large indicates the classification was entirely carried out by the network, NASNet-Large-SVM means the image features were extracted by the pre-trained network and used for training the SVM to carry out the classification task, NASNet-Large-FRP-LSTM is the proposed model that uses the pre-trained network for feature extraction, constructs the FRPs of the flattened pretrained CNN-based features, and adopts the LSTM for classifying the FRPs as multifeatured time series. Similar meanings apply for DenseNet-201, DenseNet-201-SVM, and DenseNet-201-FRP-LSTM; and ResNet-101, ResNet-101-SVM, and ResNet-101-FRP-LSTM. The results obtained from the GLCM-SVM are the same as those from DenseNet and ResNet. GooLeNet and AlexNet yielded the same values of the performance measures, where the accuracy (62.50%) is lower than the GLCM-SVM (75%). While the classification accuracy obtained from NASNet-Large and NASNet-Large-FRP-LSTM as well as DenseNet-201 and DenseNet-201-FRP-LSTM are the same (75%), ResNet-101-FRP-LSTM achieved higher accuracy (87.50 %), which is also the best rate among other 11 models. The ResNet-101-FRP-LSTM model also provided the best results for precision (83%) and $F_{1}$ score (0.91). The SVM-based classification performed poorly with features extracted from the NASNet-Large (accuracy =37.50%) and ResNet-101 (accuracy =50.00%).

**TABLE 2:** Performance Measures for 10-fold Cross-Validation Among Different Classification Models, Where NASNet, DenseNet, and ResNet are Short Names for NASNet-Large, DenseNet-201, and ResNet-101, Respectively

Model	%ACC	%SEN	%SPE	%PRE	$F_{1}$
GLCM-SVM	75.00	100.00	33.33	71.43	0.83
GoogLeNet	62.50	100.00	0.00	62.50	0.77
AlexNet	62.50	100.00	0.00	62.50	0.77
NASNet	75.00	80.00	66.67	80.00	0.80
NASNet-SVM	37.50	60	0.00	50.00	0.55
NASNet-FRP-LSTM	75.00	100	33.33	71.43	0.83
DenseNet	75.00	100.00	33.33	71.43	0.83
DenseNet-SVM	75.00	100.00	33.33	71.43	0.83
DenseNet-FRP-LSTM	75.00	100.00	33.33	71.43	0.83
ResNet	75.00	100.00	33.33	71.43	0.83
ResNet-SVM	50.00	80.00	0.00	57.14	0.67
ResNet-FRP-LSTM	87.50	100.00	66.67	83.33	0.91

Table 3 shows the time complexity of the 12 classification models, which produced the results shown in Table 2, required by a CPU@2.50GHz. Because of its large size, the training of NASNet-Large took relatively much more computational time, which is 5890 and 1120 times longer than the training of a linear SVM and bidirectional LSTM, respectively. Training the proposed approach (NASNet-Large-FRP-LSTM) is only about 5 times longer than a linear SVM. Similar comparisons of time complexity can be drawn for the other two groups of classification methods: DenseNet-201, DenseNet-201-SVM, and DenseNet-201-FRP-LSTM; and ResNet-101, ResNet-101-SVM, and ResNet-101-FRP-LSTM. Because of its shallower depth, ResNet-101 training required the least computational time. However, both the training time and total running time for ResNet-101-FRP-LSTM were longer than those for DenseNet-201-FRP-LSTM. The training time taken by either GooLeNet or AlexNet was a few times less than the other three pre-trained CNNs. The GLCM-SVM required a longer running time than the ResNet-101-SVM mainly due to the extraction of the GLCM features. Such time saving suggests an advantage of feature extraction from the ResNet-101.

**TABLE 3:** Time Complexity Among Different Classification Models for 10-Fold Cross-Validation on a Single CPU, Where NASNet, DenseNet, and ResNet are Short Names for NASNet-Large, DenseNet-201, and ResNet-101, Respectively. TT = Training Time in Seconds, TRT = Total Running Time in Seconds, and #P = Number of Parameters in Millions

Model	TT (sec)	TRT (sec)	#P (MM)
GLCM-SVM	4	91	
GooLeNet	540	589	7.0
AlexNet	360	399	61.0
NASNet	23571	23583	88.9
NASNet-SVM	4	183	88.9
NASNet-FRP-LSTM	21	265	88.9
DenseNet	1626	1632	20.0
DenseNet-SVM	3	51	20.0
DenseNet-FRP-LSTM	30	125	20.0
ResNet	1401	1407	44.6
ResNet-SVM	3	43	44.6
ResNet-FRP-LSTM	70	141	44.6

Table 3 also shows the numbers of parameters of the pretrained CNNs (the lower the better). In scientific computing, the counting of floating point operations (FLOPs) has been used to compare the computational complexity between algorithms (the lower the better). Both FLOPs and FLOPS (floating point operations per second) are adopted for different purposes. While FLOPs are used to quantify how many operations are needed to execute a computer algorithm, FLOPS (the higher the better or faster) are used for measuring the computational power of given hardware. FLOPS vary substantially between different microprocessor architectures [54].

It has been reported that the measure of FLOPs is no longer the governing factor in execution speed on modern computers [55] and becomes obsolete [56] as openly discussed in the community of numerical analysis [57]. In fact, the function for counting FLOPs is no longer available in updated versions of MATLAB. The AI community is seeking more accurate models for comparing algorithm efficiency [54]. It should be pointed out that total running times required by a pretrained CNN are different for using data of various sizes. However, measures of computer performance using ResNet-101, DenseNet-201, and NASNet-Large by means of FLOPs were reported in literature. For example, using the ImageNet database [30], total FLOPs for ResNet-101, DenseNet-201, and NASNet-Large are $8 \times 10^{9}$ [58], $4 \times 10^{9}$ [58], and $30 \times 10^{9}$ [59], respectively. These FLOPs show the most and least favorable nets among the three are DenseNet-201 and NASNet-Large, respectively.

The above analyses suggest the usefulness of the proposed approach for classifying IHC images of RhoB expression on NATs of rectal-cancer patients who took pre-operative radiotherapy. Such classification results obtained from the proposed approach not only indicate RhoB protein is a potential prognosis biomarker of rectal cancer, but are also helpful for clinical decision-making if a patient with rectal cancer should be recommended for post-operative radiotherapy to reduce local recurrence or other alternative treatments, depending on the predicted survival of the patient.

## IV. Conclusion

The foregoing sections have presented the proposed CNN-FRP-LSTM approach that extracts features of IHC images from a pre-trained CNN, transforms them into FRPs, and then uses the transformed data as multifeatured time series for classification by an LSTM network. The results illustrate that the CNN-FRP-LSTM models are more favorable in terms of the combination of accuracy and computational complexity than other classification methods for predicting the 5-year survival of rectal-cancer patients using IHC images of RhoB expression on NATs. Such promising results encourage further study of the proposed approach by exploring other pre-trained CNN models and optimal parameters for constructing FRPs of the extracted deep-learning features.

A future investigation of the proposed AI approach is to use RhoB-expression tissues of healthy subjects, tumors, and NATs to discover the predictive power of the three tissue types and molecular changes associated with cancer. Such applications of AI and machine-learning methods can be helpful for posing new questions and validating hypotheses.

### Data and Code Availability

IHC data and Matlab codes are available at the Tuan D. Pham’s personal website: https://sites.google.com/ view/tuan-d-pham/codes, under the name “Rectal-cancer NATs”.

### Author Contributions

Tuan D. Pham: Conceptualized the study, developed the AI approach, and wrote the article; Vinayakumar Ravi: Assisted in the AI implementation; Chuanwen Fan and Bin Luo: organized the database; and XFS: Conceptualized the study. All authors analyzed the results and approved the submission.
